# The role of family, school and neighbourhood in explaining inequalities in physical activity trajectories between age 9 and 18

**DOI:** 10.1016/j.ssmph.2022.101216

**Published:** 2022-08-28

**Authors:** Olivia McEvoy, Frances Cronin, Ross Brannigan, Debbi Stanistreet, Richard Layte

**Affiliations:** a Department of Sociology, School of Social Sciences and Philosophy, Trinity College, Dublin, Ireland; b Department of Public Health and Epidemiology, Royal College of Surgeons in Ireland, Ireland

**Keywords:** Health inequalities, Physical activity, Family, School, Neighbourhood, Ireland, Longitudinal data, Social determinants of health

## Abstract

Differentials in physical activity (PA) between social and economic groups has been shown to contribute significantly to social gradients in health and life expectancy, yet relatively little is known about why differentials in PA emerge. This paper uses longitudinal data on a nationally representative sample of 6,216 young people aged between 9 and 18, from Ireland, to measure the role of family, school and neighbourhood level factors in accounting for differentials in PA trajectories between groups of young people, defined by level of maternal education, whilst adjusting for the individual characteristics of the young person (sex, age, personality, body mass index and health-status). Levels of PA fall significantly across the sample between 9 and 18, and the decline in PA is larger for the children of lower educated mothers. We find a clear gradient in PA at each age by maternal education for both males and females. Descriptive analyses found social gradients in the majority of our risk factors. Using multi-level, linear spline regression models to decompose differentials between groups, we find that family-level mechanisms account for the biggest proportion of the differential in PA for both males (50.8%) and females (35.1%). Differences in income across maternal education categories accounted for 24.1% of the differential for males and 14.7% among females, making it the second most effective mechanism in explaining the social patterning of PA. Neighbourhood-level processes resulted in a modest reduction in the same differential, while school level processes had the effect of equalising differences in PA across maternal education groups.

## Highlights

• The potential social determinants of inequalities in physical activity were outlined.
• The role of family, school and neighbourhood in explaining these inequalities was quantified.
• The cumulative effect of social environment was observed over a 10-year period.
• Family-level characteristics were identified as the most effective explanatory mechanism.
• Highly educated mothers were observed to be enrolling their children in organised sport.

## 1 Introduction

Non-communicable diseases (NCDs) are the largest cause of mortality globally, accounting for 71% of all deaths, despite the common modifiable risk factors (tobacco and alcohol use, unhealthy diet and physical inactivity) being well-established (World Health Organisation, 2021). The inverse association between social and economic position (SEP) and NCDs, known as the social gradient in health, is one of the most robust findings in epidemiology. Research suggests that the social patterning of health behaviours is a major determinant of the social gradient in health, with robust evidence showing that these behavioural factors are also differentially distributed by SEP (Petrovic et al., 2018).

Less is known about what Marmot (2018) has referred to as “the causes of the causes” of health inequalities, that is *why* health behaviours vary across SEP in a structured manner. Models of behaviour change often focus on the role of knowledge, beliefs and intentions as the main determinants of poor health behaviours (Ogden, 2016) but there is a long-standing literature that argues that health behaviours must be understood in their social and economic context and viewed as a response to conditions imposed by social structures (House, 2002; Marmot et al., 1997). It has also been argued that adult health behaviours need to be viewed through a life-course perspective, as the cumulative response of individuals and groups to circumstances in which they live (Lynch et al., 1997).

In response, this paper focuses on social gradients in physical activity (PA), specifically the role of child environment in shaping the young person's PA trajectory from middle childhood to early adulthood (from age 9 to 17/18). Whilst adjusting for individual differences in personality BMI and health status, we empirically assess the role of the child's family, school and neighbourhood environment in shaping PA over a crucial period of development. Social gradients in PA have been consistently found among adolescents (Inchley et al., 2016, 2020) and research has shown that there is a sharp decline in PA during adolescence between 11 and 15yrs (Dumith et al., 2011; Farooq et al., 2018; Rovio et al., 2018). Adolescence has been described as a critical period for health behaviour formation, with habits established during adolescence likely to continue into adulthood (Alberga et al., 2012).

### 1.1 Family-level mechanisms

The family is a primary site of socialisation and both the behaviours of parents and the specific parenting practices that parents adopt can have an influence on children's levels of PA. Parental level of PA can act as a role model for their children, but parents also have an important ‘gate-keeping’ role by introducing their child to their own sporting interests. There is a proven link between having a parent that plays sport the likelihood that their child will play sport (Lunn, 2006; McMinn et al., 2008).

Differences in parenting practices, across households, are also a potential pathway for the intergenerational transmission of health behaviours. Lareau's (2011) ethnographic research demonstrated that high SEP parents are more likely to engage in what she calls “concerted cultivation” by enrolling their children in various enrichment activities (e.g. learning an instrument, organised team sport), whereas lower SEP parents are more likely to adopt a strategy of “natural growth” where young people get more discretion and less of their free time is structured. Unstructured and unsupervised time is an opportunity for children to engage in an array of tempting sedentary pursuits that compete for young people's time, and have been shown to lead to physically inactive lifestyles (Pearson et al., 2014).

### 1.2 School-level mechanisms

Young people from different social and economic backgrounds are not randomly distributed across schools, and schools vary in terms of the type and amount of funding they have access to. Self-reported principal data, in the Irish context, demonstrates that some schools have access to better quality and a greater variety of school sporting facilities (Fahey et al., 2005; Woods et al., 2010, 2019). Variation in sport facilities may contribute to explaining the social gradients in PA, given the long-term relationship between attending a school with inadequate sports facilities and the likelihood of being physically inactive in adulthood (Black et al., 2019).

Mode of transport to and from school may contribute to gradients in PA. Children with an active commute can increase their PA levels, and improve their fitness, without the need for planned sports or activities but little is known about the distribution of active commute across SEP groups (Heelan et al., 2005; Murtagh et al., 2016).

### 1.3 Neighbourhood-level mechanisms

The emergence of socio-ecological models of health behaviours (Sallis et al., 2015) has led to an abundance of literature successfully demonstrating the extent to which a young person's physical environment impacts their level of PA (McGrath et al., 2015; Sterdt et al., 2013). A number of different dimensions of local area have been shown to impact on PA. Lower social and economic groups may be more likely to be confronted with these barriers to PA (Handy et al., 2002). This paper will focus on examining the role of neighbourhood safety and neighbourhood social and physical disorder, such as crime, public loitering, vandalism, graffiti and abandoned buildings, in reducing levels of PA, particularly among low SEP female adolescents as the perceived risk to physical safety may be heightened in this group (Dulin-Keita et al., 2013).

### 1.4 Parental resources: education & income

The association of education with health behaviours is now well established with lower education associated with a greater prevalence of smoking, higher alcohol consumption, a less healthy diet and less physical activity (Cavelaars et al., 2000; Droomers et al., 1999; Gidlow et al., 2006). Parental and particularly maternal higher education is associated with greater health literacy and self-efficacy, both of which are influential in shaping the probability of children engaging in health-promoting behaviours (Prickett & Augustine, 2016).

The financial resources available to a family may have a direct bearing on the participation of the young person in PA. Lower income and savings may directly restrict the ability of the family to pay for the young person's membership of sports clubs, buy sports equipment/clothing or provide transport to sports events. Previous research has shown a clear effect of family income on the level and type of PA that adolescents participate in and whether or not they are members of sports clubs (Kantomaa et al., 2007).

## 2 Material & methods

### 2.1 Sample

Growing Up in Ireland (GUI) is a nationally representative cohort study of young people living in the Republic of Ireland. GUI commenced in 2007/2008 with 8,568 participating children aged 9 (T1). Data was subsequently collected when participants were aged 13 (T2; 2011/2012) with an 88% retention rate (n = 7,525), and aged 17/18 (T3; 2015/2016) with a 73% retention rate (n = 6,216) (Murray et al., 2010). The sample was selected through a two-stage clustered sampling method within the Irish primary school system, whereby the school was the primary sampling unit and the age eligible young people attending the school were the secondary units. Probability proportionate to size (PPS) sampling method was used to select a representative sample of 910 primary schools (82% response rate) and from these 8,568 (57% response rate) families agreed to participate. Information was collected from participants parents, teachers and school principals by trained interviewers.

The loss of participants to follow-up raises concerns that the pattern of attrition may be selective and thus introduce systematic bias into our analyses. The data were reweighted (Murphy et al., 2018; Thornton et al., 2010) for analyses to take account of the original sample error and subsequent attrition using a range of factors and a minimum distance algorithm. The large sample sizes, the probability samples and the calibration to ensure representativeness mean that the results can be generalised to the population. The final reweighted sample is representative of young people who were residing in Ireland at 9 years of age in 2006, and who continued to live in Ireland at 17/18 years of age in 2016. Further details on the design and reweighting procedures are available elsewhere (Murphy et al., 2018; Thornton et al., 2010).

**Fig. 1:**
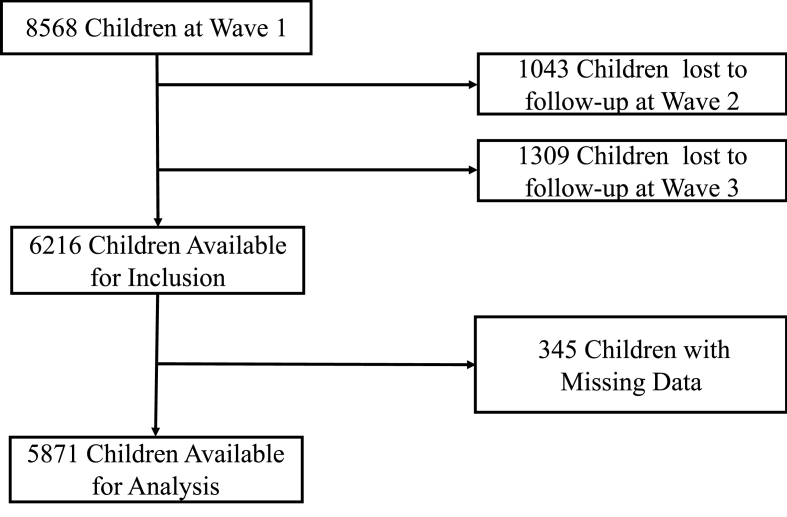
Sample exclusion criteria and final case base for analysis.

Analyses were restricted to participants who had available data at all three waves on our variables of interest. This reduced our sample to 5,871 (see Fig. 1). All available data from all eligible participants were used under a missing-at-random assumption.

### 2.2 Measures

#### 2.2.1 Dependent variable

PA levels were calculated using the Godin-Shephard Leisure Time Exercise Questionnaire (Godin & Shephard, 1985) at all three timepoints. The primary caregiver (PCG) reported the number of days out of the previous 14 that the young person had engaged in “hard” or “light” exercise for at least 20 min at T1 & T2, and the young person (YP) reported at T3. Hard exercise was defined as “enough to make him/her breathe heavily and make his/her heart faster”. Light exercise was defined as “not hard enough to make him/her breathe heavily and make his/her heart beat fast” (Godin & Shephard, 1985). The Godin-Shephard has previously demonstrated concurrent validity with measures of maximum oxygen intake and muscular endurance (Godin et al., 1986), construct validity (Godin & Shephard, 1985; Zelener & Schneider, 2016), and test-retest reliability (Sallis et al., 1993; Zelener & Schneider, 2016).

#### 2.2.2 Individual-level control variables

The YP's personality was measured at T2 by PCG report using the Ten Item Personality Inventory (TIPI) and was assumed to be fixed across the three timepoints. TIPI consists of five subscales: agreeableness, extraversion, conscientiousness, emotional stability, and openness to experience (Gosling et al., 2003). The significant associations between personality and PA, specifically agreeableness conscientiousness and extraversion are evidenced elsewhere (Rhodes & Smith, 2006; Wilson & Dishman, 2015). The PCG reported on whether or not the YP had a chronic illness (“yes”/“no”) at all three timepoints. This variable was included to control for instances where participants could not be active for health reasons. BMI was recorded at all three timepoints by trained interviewers, who carried out height and weight measurements. Standard sex and age specific BMI cut-off points were used to classify participants as “normal weight,” “overweight” “obese” or “missing” (Cole et al., 2000). BMI has a reciprocal relationship with PA, where physical inactivity can lead to increased BMI but increased BMI has also been shown to be an independent significant negative predictor of PA (Pietiläinen et al., 2008). The final control variable was reported by the PCG at T1 indicating whether the YP lives in an “urban” or “rural” area, rural was defined as “open country” or “a village” with a population of less than 1,500 persons (Central Statistics Office (CSO) (2019). 31.4% of Ireland's population live in a rural area, which is above average within the EU making the urban rural distribution an important feature of Irish life, especially for health behaviour research as rural adolescents were more likely to be obese than those in urban areas (Li et al., 2017).

#### 2.2.3 Independent variable: maternal education

Maternal highest reported qualification was coded using the International Standard Classification of Education (UNESCO Institute for Statistics, 2012 2011) and collapsed into four groups: lower secondary (junior certificate) (ISCED 0–2), upper secondary (leaving certificate) (ISCED 3), post-secondary (diploma) (ISCED 4 & 5) and tertiary, i.e. bachelor's degree or above (ISCED 6–8).

#### 2.2.4 Income

The PCG reported on each household income from all household members at all three timepoints. In order to make meaningful comparisons between households on their income, household size and structure was taken into account by assigning a weight to each household member to create an ‘equivalised’ total disposable income (after tax and welfare transfers). The first adult is assigned a weight of 1 with a weight of 0.66 allocated to all subsequent adults and 0.33 to each child (14 years or less). These weightings are identical to those used for the Irish official income poverty line (Roantree et al., 2021). Equivalised income was ranked and transformed into income quintiles with the addition of a category for missing income.

#### 2.2.5 Family-level variables

The YP's attendance (“yes”/“no”) at several activities was reported by the PCG at T1 and the YP at T2 & T3. Activities included: dance, music, drama, art, youth club, homework club, scouts, guides, and organised sport (with an instructor). With the exception of organised sport, the activities were combined into one “enrichment activities” variable. The combination of these activities is used to measure the extent to which parents practise concerted cultivation (Lareau, 2011). Organised sport was kept separate so as to not artificially inflate the importance of concerted cultivation in explaining the relationship between maternal education and PA. Minutes on a normal weekday that the YP spent doing “unstructured activities” such as watching TV, playing video games and on the computer was reported by the PCG at T1 and the YP at T2 & T3. These variables were combined. This variable is used to represent the degree to which parents practised accomplishment of natural growth, as demonstrated elsewhere (McCoy et al., 2012). PCG's also reported the regularity of their own exercise at T2. They classified themselves as “very physically active,” “fairly physically active,” “not very physically active” or “not at all physically active.”

#### 2.2.6 School-level variables

School principals reported on the adequacy of the school's sporting faculties in primary (T1) and secondary (T2 & T3) school. They indicated whether they were “poor,” “fair,” “good” or “excellent,” when compared to other schools in Ireland. The categories were recoded to combine conceptually similar categories, and resulted in two new categories: “poor/fair” and “good/excellent.” The PCG reported how their child usually travelled to primary (T1) and secondary (T2 & T3) school. There were six response categories: “walks” “public transport” “school bus” “car” “rides a bicycle” or “other.” Walking or cycling was classified as active commuting, due to the small number of cyclists, both bus categories (public and school) were also combined and the “other” category was excluded from analyses.

#### 2.2.7 Neighbourhood-level variables

The PCG reported on their perceived safety of their neighbourhood at all three timepoints. Using a four-point scale from strongly agree to strongly disagree they reported on whether: (1) it is safe to walk alone in this area after dark (2) it is safe for children to play outside during the day in this area (3) there are safe parks, playgrounds and play spaces in this area. Parental-perceived neighbourhood safety is significantly associated with encouraging outdoor physical activity (Kimbro et al., 2011; Kimbro & Schachter, 2011; Nicksic et al., 2018) and less screen time (Hunter et al., 2020). The PCG also reported on the quality of the neighbourhood, using a four-point scale they stated how common the following occurrences were: (1) rubbish and litter lying about (2) homes and gardens in bad condition (3) vandalism and deliberate damage to property (4) people being drunk or taking drugs in public. Lower SEP families were much more likely to report unfavourable physical conditions in their local neighbourhood (Williams et al., 2009).

### 2.3 Analysis strategy

First, we carried out descriptive statistics (frequencies and percentages, means and standard deviations) on our dependent and potential mediating variables by maternal education categories at each timepoint. Pearson's correlation coefficient, independent sample t-tests and one-way ANOVAs, where appropriate, were used to examine statistically significant differences across maternal education categories. Levene's statistic was used to determine that the assumption of the equality of variance was met, and if the ANOVAs were valid. Where the assumption of the equality of variance was not met Welch's F was used instead.

Multi-level linear spline models (Howe et al., 2016), with appropriate study weights, were used to estimate PA trajectory differentials by maternal education level between ages 9 and 17/8. To account for the nesting of PA measures within individuals, each model includes a random intercept (μ0_j_) for each young person, which follows a bivariate normal distribution and measurement error is explicitly modelled over time (e0_ij_). The random intercepts model for linear change in PA can be written as:

3 Y_ij_ = β0 + β1 x (age)_ij_ + β2 x (Maternal education)j + β

x (Maternal education)_i_ x (Age) _ij_ + μ0_j_ + e0_ij_

Here, β0, β1, β2 and β3 were “fixed” coefficients, which represent the average intercept across the sample and the average slopes for age, maternal education plus the interaction of age and maternal education. μ0_j_ is the “random” coefficient, which represents the individual's deviation from the average intercept. e0_ij_ represents the observation-level measurement error.

We quantified the role of different groups of predictors in accounting for the maternal education differential by calculating the change in β1+β2+β3 with the addition of each group of predictors to the model. Model 2 (baseline and controls) was our reference point. Theoretical justification was used to assign variables to each of the models (income, family, school and neighbourhood). Models were stratified by sex. All analyses were performed in Stata/SE 16. P-values less than 0.05 were considered statistically significant.

## 3 Results

### 3.1 Maternal education and PA

**Graph 1:**
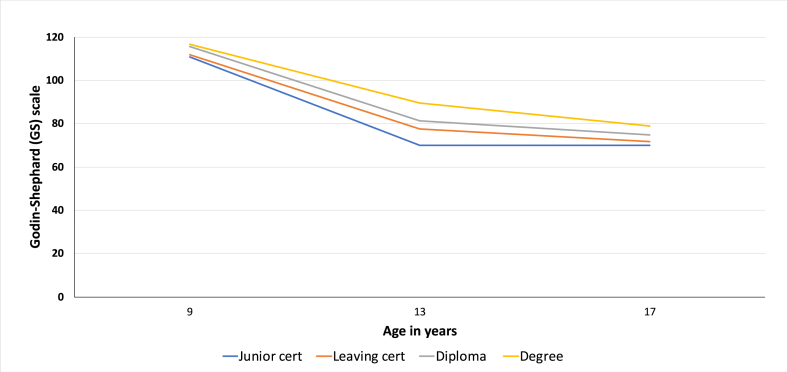
Levels of PA on the GS scale by maternal education categories.

Fig. 2 (see graph below) demonstrates the clear social gradient by maternal education in levels of PA, measured using the GS scale. The differences in average levels of PA between the four maternal education groups were statistically significant (p < 0.001) at age 9, 13 and 17/18. The differential between the highest and lowest maternal education group is widest at age 13 (19.6) compared to age 9 (5.9) and age 17/18 (8.9). Graph 1 also demonstrates the clear age gradient in levels of PA over the 10 year period, where activity levels were highest at age 9 (114.0), decreasing at age 13 (80.8) and lowest at age 17/18 (74.1).

### 3.2 Maternal education and family, school and neighbourhood-level mediators

Table 1 (below) demonstrates the social patterning of our variables of interest. Families with low levels of maternal education were also likely to have low levels of income. The family-level mediating variables show a clear social patterning: young people whose mothers have lower levels of education report more adverse behaviours and outcomes at each timepoint. Lower levels of maternal education were associated with YP's non-participation in organised sport and non-attendance at other structured activities, and spending more time doing unstructured activities. Highly educated mothers also tended to be “very” or “fairly” physically active themselves, compared to “not very physically active” and “not physically active.”

**Table 1:** Mediating variables by maternal education categories.

	Junior Cert. (N = 942)	Leaving Cert. (N = 1909)	Diploma (N = 1587)	Degree (N = 1778)	Total (N = 6216)	p-value
**Income (age 9)**						<0.001
lowest	259 (27.5%)	210 (11.0%)	119 (7.5%)	57 (3.2%)	645 (10.4%)	
2^nd^	240 (25.5%)	380 (19.9%)	212 (13.4%)	149 (8.4%)	981 (15.8%)	
3^rd^	181 (19.2%)	438 (22.9%)	316 (19.9%)	232 (13.0%)	1167 (18.8%)	
4^th^	128 (13.6%)	405 (21.2%)	430 (27.1%)	406 (22.8%)	1369 (22.0%)	
highest	71 (7.5%)	329 (17.2%)	398 (25.1%)	814 (45.8%)	1612 (25.9%)	
*missing*	63 (6.7%)	147 (7.7%)	112 (7.1%)	120 (6.7%)	442 (7.1%)	
**Income (age 13)**						<0.001
lowest	259 (29.2%)	311 (16.7%)	163 (10.6%)	93 (5.3%)	826 (13.7%)	
2nd	237 (26.7%)	320 (17.2%)	224 (14.5%)	139 (8.0%)	920 (15.2%)	
3^rd^	170 (19.2%)	381 (20.4%)	275 (17.8%)	223 (12.8%)	1049 (17.4%)	
4^th^	100 (11.3%)	427 (22.9%)	394 (25.5%)	409 (23.4%)	1330 (22.0%)	
highest	56 (6.3%)	288 (15.4%)	373 (24.2%)	768 (44.0%)	1485 (24.6%)	
*missing*	64 (7.2%)	138 (7.4%)	114 (7.4%)	113 (6.5%)	429 (7.1%)	
**Income (age 17)**						<0.001
lowest	270 (28.7%)	249 (13.0%)	149 (9.4%)	72 (4.0%)	740 (11.9%)	
2nd	228 (24.2%)	354 (18.5%)	229 (14.4%)	137 (7.7%)	948 (15.3%)	
3^rd^	164 (17.4%)	412 (21.6%)	290 (18.3%)	249 (14.0%)	1115 (17.9%)	
4^th^	116 (12.3%)	369 (19.3%)	345 (21.7%)	418 (23.5%)	1248 (20.1%)	
highest	64 (6.8%)	316 (16.6%)	394 (24.8%)	744 (41.8%)	1518 (24.4%)	
*missing*	100 (10.6%)	209 (10.9%)	180 (11.3%)	158 (8.9%)	647 (10.4%)	
**Maternal PA**						<0.05
Very physically active	178 (20.1%)	357 (19.1%)	316 (20.5%)	333 (19.1%)	1184 (19.6%)	
Fairly physically active	472 (53.3%)	1067 (57.2%)	850 (55.1%)	1028 (58.9%)	3417 (56.6%)	
Not very physically active	188 (21.2%)	383 (20.5%)	331 (21.5%)	332 (19.0%)	1234 (20.4%)	
Not at all physically active	48 (5.4%)	58 (3.1%)	46 (3.0%)	52 (3.0%)	204 (3.4%)	
**Children's activities (age 9)**						<0.001
0	430 (45.6%)	701 (36.7%)	536 (33.8%)	453 (25.5%)	2120 (34.1%)	
1	374 (39.7%)	930 (48.7%)	807 (50.9%)	1041 (58.5%)	3152 (50.7%)	
2+	138 (14.6%)	278 (14.6%)	244 (15.4%)	284 (16.0%)	944 (15.2%)	
**Children's activities (age 13)**						<0.001
0	378 (42.8%)	777 (41.8%)	617 (40.2%)	609 (35.0%)	2381 (39.5%)	
1	351 (39.7%)	739 (39.7%)	616 (40.1%)	744 (42.8%)	2450 (40.7%)	
2+	155 (17.5%)	345 (18.5%)	303 (19.7%)	387 (22.2%)	1190 (19.8%)	
**Children's activities (age 17)**						<0.001
0	605 (64.2%)	1194 (62.5%)	956 (60.2%)	997 (56.1%)	3752 (60.4%)	
1	247 (26.2%)	567 (29.7%)	491 (30.9%)	596 (33.5%)	1901 (30.6%)	
2+	90 (9.6%)	148 (7.8%)	140 (8.8%)	185 (10.4%)	563 (9.1%)	
**SBSB (age 9)**						<0.001
mean (SD)	205.4 (122.2)	186.2 (104.5)	173.6 (103.5)	154.4 (100.2)	176.8 (107.3)	
**SBSB (age 13)**						<0.001
mean (SD)	99.9 (63.8)	90.7 (57.2)	86.5 (60.7)	72.7 (52.8)	85.8 (58.6)	
**SBSB (age 17)**						<0.001
mean (SD)	271.8 (141.5)	251.7 (135.9)	229.6 (128.5)	210.8 (123.6)	236.7 (132.9)	
**Organised sport (age 9)**						<0.001
No	320 (34.0%)	391 (20.5%)	270 (17.0%)	260 (14.6%)	1241 (20.0%)	
Yes	621 (66.0%)	1514 (79.5%)	1317 (83.0%)	1518 (85.4%)	4970 (80.0%)	
**Organised sport (age 13)**						<0.001
No	267 (30.6%)	417 (22.5%)	282 (18.4%)	224 (13.0%)	1190 (19.9%)	
Yes	605 (69.4%)	1434 (77.5%)	1247 (81.6%)	1503 (87.0%)	4789 (80.1%)	
**Organised sport (age 17)**						<0.001
No	494 (52.4%)	824 (43.2%)	628 (39.6%)	606 (34.1%)	2552 (41.1%)	
Yes	448 (47.6%)	1085 (56.8%)	959 (60.4%)	1172 (65.9%)	3664 (58.9%)	
**Primary school commute**						<0.001
active commute	329 (35.2%)	545 (28.7%)	366 (23.3%)	427 (24.3%)	1667 (27.1%)	
bus	147 (15.7%)	278 (14.7%)	230 (14.7%)	241 (13.7%)	896 (14.6%)	
car	458 (49.0%)	1074 (56.6%)	973 (62.0%)	1090 (62.0%)	3595 (58.4%)	
**Secondary school commute**						<0.001
active commute	246 (27.8%)	343 (18.5%)	264 (17.2%)	313 (18.2%)	1166 (19.4%)	
bus	282 (31.9%)	587 (31.6%)	480 (31.2%)	495 (28.8%)	1844 (30.7%)	
car	356 (40.3%)	927 (49.9%)	793 (51.6%)	912 (53.0%)	2988 (49.8%)	
**Primary school sports facilities**						0.255
poor/fair	392 (43.0%)	839 (45.4%)	661 (43.0%)	782 (45.9%)	2674 (44.5%)	
good/excellent	520 (57.0%)	1011 (54.6%)	876 (57.0%)	923 (54.1%)	3330 (55.5%)	
**Secondary school sports facilities**						0.165
poor/fair	259 (31.1%)	569 (31.8%)	446 (30.2%)	480 (28.4%)	1754 (30.3%)	
good/excellent	574 (68.9%)	1218 (68.2%)	1033 (69.8%)	1209 (71.6%)	4034 (69.7%)	
**Disorder in Local Area (age 9)**						<0.001
mean (SD)	2.0 (0.7)	1.8 (0.6)	1.8 (0.6)	1.7 (0.5)	1.8 (0.6)	
**Disorder in Local Area (age 13)**						<0.001
mean (SD)	1.8 (0.7)	1.7 (0.6)	1.6 (0.6)	1.6 (0.5)	1.7 (0.6)	
**Disorder in Local Area (age 17)**						<0.001
mean (SD)	2.1 (0.8)	2.0 (0.7)	1.9 (0.6)	1.9 (0.6)	2.0 (0.7)	
**Safety in Local Area (age 9)**						<0.001
mean (SD)	2.8 (0.6)	2.9 (0.6)	2.9 (0.6)	3.0 (0.6)	2.9 (0.6)	
**Safety in Local Area (age 13)**						<0.001
mean (SD)	2.9 (0.7)	2.9 (0.6)	2.9 (0.6)	3.0 (0.6)	2.9 (0.6)	
**Safety in Local Area (age 17)**						<0.001
mean (SD)	2.8 (0.7)	2.9 (0.7)	2.9 (0.6)	2.9 (0.6)	2.9 (0.6)	

School-level mediating variables do not follow a clear social patterning where low levels of maternal education were associated with more adverse behaviours and outcomes. In terms of commuting to school, participants whose mothers had a lower or higher secondary qualification education were more likely to be active commuters, a positive health behaviour, than participants whose mothers had tertiary education. However, participants whose mothers had low levels of education were still less likely to travel to school by car and more likely to get the bus, when compared to participants whose mothers had higher levels of education. There were no significant differences in the perceived adequacy of school sporting facilities across maternal education groups.

The neighbourhood-level mediators, perceived neighbourhood disorder and perceived neighbourhood safety, were both significantly (p < 0.001) associated with maternal education. Young people whose mothers had lower levels of education being more likely to live in areas with higher levels of social disorder and lower levels of neighbourhood safety.

### 3.3 Multivariate analysis

Table 2, Table 3 (below) show the results of multi-level linear spline regression models predicting levels of PA for males and females respectively. In the base model, young people whose mothers have achieved lower or higher secondary education were significantly less likely to be PA than young people whose mothers who have achieved tertiary education. There were no statistically significant differences in levels of PA between young people whose mothers have a post-secondary level qualification (“diploma”) and those whose mothers have tertiary education (“degree”). In Model 2, the individual-level controls were added to the base model.

**Table 2:** Results from multi-level linear spline regression models predicting levels of PA (males).

	Model 1: Base	Model 2: Controls	Model 3: Income	Model 4: Family	Model 5: School	Model 5: Neighbourhood	Model 5: Fully adjusted
	PA (s.e)	PA (s.e)	PA (s.e)	PA (s.e)	PA (s.e)	PA (s.e)	PA (s.e)
**Maternal education level**							
junior cert. (vs. degree)	−8.59*** (2.24)	−7.62*** (2.19)	−5.63* (2.24)	−3.84 (2.14)	−7.90*** (2.20)	−7.50*** (2.20)	−3.05 (2.19)
leaving cert. (vs. degree)	−7.55*** (2.09)	−6.14** (2.05)	−4.91* (2.06)	−5.55** (1.99)	−6.25** (2.05)	−6.01** (2.05)	−4.94*(2.00)
higher diploma (vs. degree)	−2.37 (2.43)	−1.07 (2.38)	−0.29 (2.38)	−0.23 (2.31)	−1.16 (2.38)	−0.98 (2.38)	0.02 (2.31)
**Age in years**							
13 (vs. 9)	−27.19*** (2.15)	−27.28*** (2.15)	−27.48*** (2.15)	−26.23*** (2.16)	−27.07*** (2.16)	−27.27*** (2.15)	−26.28*** (2.17)
17/18 (vs.9)	−34.86*** (2.15)	−35.19*** (2.15)	−35.40*** (2.15)	−31.56*** (2.17)	−34.99*** (2.15)	−35.19*** (2.15)	−31.72***(2.18)
**Maternal Education#Age**							
junior cert.#13	−6.83* (2.82)	−6.44* (2.82)	−6.18* (2.82)	−5.48 (2.83)	−6.19* (2.82)	−6.40* (2.82)	−4.99 (2.83)
junior cert.#17/18	5.79* (2.83)	6.16* (2.83)	6.40* (2.83)	7.04* (2.84)	6.42* (2.83)	6.16* (2.83)	7.56** (2.84)
leaving cert.#13	−2.91 (2.64)	−3.06 (2.63)	−2.86 (2.63)	−0.63 (2.64)	−2.79 (2.63)	−3.20 (2.63)	−0.29 (2.64)
leaving cert.#17/18	1.46 (2.63)	1.86 (2.63)	2.00 (2.63)	3.75 (2.64)	2.14 (2.63)	1.75 (2.63)	4.02 (2.64)
diploma#13	−2.85 (3.07)	−3.50 (3.07)	−3.19 (3.07)	−2.20 (3.08)	−3.26 (3.07)	−3.65 (3.07)	−1.80 (3.08)
diploma#17/18	0.64 (3.08)	0.42 (3.07)	0.51 (3.07)	0.52 (3.08)	0.69 (3.07)	0.30 (3.07)	0.71 (3.08)
**Child chronic illness**							
no (vs. yes)		−5.93*** (1.39)	−5.78*** (1.38)	−4.62*** (1.35)	−5.85*** (1.39)	−5.84*** (1.39)	−4.37**(1.34)
**Personality**							
agreeableness		−1.30** (0.46)	−1.28** (0.46)	−1.10** (0.43)	−1.33** (0.46)	−1.31** (0.46)	−1.06*(0.42)
conscientiousness		0.83 (0.43)	0.86* (0.42)	0.24 (0.40)	0.84* (0.43)	0.83 (0.43)	0.30 (0.39)
emotional stability		1.97*** (0.40)	1.99*** (0.40)	1.53*** (0.37)	1.99*** (0.40)	1.98*** 0.4	1.59***(0.37)
extraversion		4.11*** (0.39)	4.06*** (0.39)	3.16*** (0.36)	4.11*** (0.39)	4.09*** (0.39)	3.15***(0.36)
openness to experience		−0.04 (0.49)	0.08 (0.49)	0.42 (0.46)	−0.02 (0.49)	−0.02 (0.49)	0.56 (0.45)
**Child BMI**							
overweight (vs normal)		−3.46** (1.12)	−3.50** (1.12)	−2.70* (1.08)	−3.50** (1.12)	−3.50** (1.12)	−2.68*(1.08)
obese (vs. normal)		−16.57*** (1.96)	−16.64*** (1.95)	−14.27*** (1.89)	−16.50*** (1.96)	−16.61*** (1.96)	−14.20***(1.89)
missing (vs. normal)		−7.88*** (2.39)	−8.01*** (2.38)	−6.72** (2.34)	−7.99*** (2.39)	−7.94*** (2.39)	−6.93**(2.34)
**Region**							
rural (vs urban)		−4.58*** (0.96)	−4.09*** (0.96)	−5.60*** (0.89)	−3.74*** (1.01)	−4.43*** (0.99)	−3.94***(0.96)

lowest (vs. highest)			−5.60*** (1.54)				−3.17*(1.49)
2nd (vs. highest)			−4.50** (1.46)				−2.08 (1.41)
3rd (vs. highest)			−6.18*** (1.42)				−4.81***(1.37)
4th (vs. highest)			−1.00 (1.36)				0.25 (1.32)
missing (vs. highest)			−0.23 (1.80)				0.68 (1.75)
**Maternal PA**							
fairly (vs. very physically active)				−1.91 (1.12)			−1.61 (1.12)
not very (vs. very physically active)				−4.73***(1.39)			−4.36**(1.39)
not (vs. very physically active)				−12.97***(2.48)			−13.04***(2.48)
**Enrichment activities**				−0.07 (0.59)			−0.1 (0.59)
**Organised sport**				20.10***(1.07)			20.04***(1.07)
**Unstructured activities**				−0.02***(0.00)			−0.02***(0.00)
**School sporting facilities**							
excellent/good (vs. poor/fair)					−0.21 (0.93)		−0.25-0.89
**School commute**							
bus (vs. active commute)					−3.77** (1.30)		−4.23***(1.25)
car (vs. active commute)					−1.38 (1.13)		−2.28*(1.09)
**Perceiveddisorder**						0.39 (0.70)	0.79 (0.69)
**Perceivedsafety**						2.13** (0.69)	1.68* (0.67)
**Constant**	122.36*** (1.71)	98.58*** (4.16)	99.54*** (4.17)	92.24*** (4.10)	99.62*** (4.21)	91.50*** (4.98)	86.59***(4.99)
**Observations**	8,494	8,494	8,494	8,494	8,494	8,494	8,494
**Number of id**	2,915	2,915	2,915	2,915	2,915	2,915	2,915
Controls	YES	YES	YES	YES	YES	YES	YES

Standard errors in parentheses.

***p < 0.001, **p < 0.01, *p < 0.05.

**Table 3:** Results from multi-level linear spline regression models predicting levels of PA (females).

	Model 1: Base	Model 2: Controls	Model 3: Income	Model 4: Family	Model 5: School	Model 5: Neighbourhood	Model 5: Fully adjusted
	PA (s.e)	PA (s.e)	PA (s.e)	PA (s.e)	PA (s.e)	PA (s.e)	PA (s.e)
**Maternal education level**							
junior cert. (vs. degree)	−5.49* (2.24)	−2.82 (2.24)	−0.69 (2.30)	1.68 (2.18)	−2.81 (2.24)	−2.34 (2.24)	2.69 (2.24)
leaving cert. (vs. degree)	−4.33* (2.16)	−3.11 (2.13)	−1.40 (2.17)	−0.95 (2.07)	−3.19 (2.13)	−2.88 (2.13)	−0.04 (2.10)
higher diploma (vs. degree)	0.24 (2.61)	1.54 (2.58)	3.01 (2.59)	2.65 (2.50)	1.58 (2.57)	1.90 (2.57)	3.67 (2.51)
**Age in years**							
13 (vs. 9)	−27.74***(2.33)	−28.04***(2.33)	−27.99***(2.34)	−27.67***(2.32)	−27.60***(2.34)	−28.31***(2.33)	−27.56***(2.33)
17/18 (vs.9)	−40.66***(2.33)	−40.87***(2.33)	−40.75***(2.33)	−34.42***(2.33)	−40.44**(2.33)	−40.71***(2.33)	−34.19***(2.34)
**Maternal Education#Age**							
junior cert.#13	−17.71*** (2.90)	−17.55*** (2.90)	−17.64*** (2.91)	−17.09*** (2.88)	−17.56*** (2.90)	−17.14***(2.90)	−16.91***(2.88)
junior cert.#17/18	−7.76** (2.90)	−7.73** (2.90)	−7.87** (2.91)	−6.88* (2.88)	−7.74** (2.90)	−7.37*(2.900	−6.75*(2.88)
leaving cert.#13	−11.02*** (2.79)	−10.83*** (2.79)	−10.99*** (2.79)	−9.99*** (2.77)	−10.64*** (2.79)	−10.83*** (2.79)	−9.94***(2.77)
leaving cert.#17/18	−5.36 (2.78)	−5.08 (2.78)	−5.40 (2.79)	−3.48 (2.76)	−4.89 (2.78)	−4.74 (2.78)	−3.31 (2.76)
diploma#13	−14.08*** (3.36)	−14.10*** (3.36)	−14.41*** (3.36)	−13.07*** (3.33)	−14.10*** (3.36)	−14.20*** (3.35)	−13.36***(3.33)
diploma#17/18	−10.90** (3.36)	−11.13*** (3.36)	−11.62*** (3.37)	−9.74** (3.34)	−11.08*** (3.36)	−10.93** (3.36)	−9.93**(3.34)
**Child chronic illness**							
no (vs. yes)		−2.29 (1.61)	−2.12 (1.61)	−2.47 (1.56)	−2.17 (1.61)	−2.01 (1.61)	−2.08 (1.56)
**Personality**							
agreeableness		−0.72 (0.44)	−0.69 (0.44)	−0.62 (0.41)	−0.74 (0.44)	−0.74 (0.44)	−0.61 (0.41)
conscientiousness		1.28** (0.41)	1.29** (0.41)	0.89* (0.38)	1.26** (0.41)	1.28** (0.41)	0.89*(0.38)
emotional stability		1.53*** (0.36)	1.52*** (0.36)	0.90** (0.34)	1.57*** (0.36)	1.41*** (0.36)	0.84*(0.34)
extraversion		2.66*** (0.38)	2.57*** (0.38)	1.69*** (0.36)	2.65*** (0.38)	2.58*** (0.38)	1.65***(0.36)
openness to experience		1.23* (0.49)	1.37** (0.49)	1.40** (0.46)	1.25* (0.49)	1.36** (0.49)	1.61***(0.46)
**Child BMI**							
overweight (vs normal)		−4.90*** (1.08)	−4.70*** (1.08)	−3.53*** (1.04)	−4.90*** (1.08)	−4.73*** (1.08)	−3.34**(1.04)
obese (vs. normal)		−9.96*** (1.67)	−9.40*** (1.67)	−7.92*** (1.61)	−9.84*** (1.67)	−9.85*** (1.67)	−7.48***(1.60)
missing (vs. normal)		−3.48 (2.14)	−3.57 (2.14)	−1.64 (2.09)	−3.46 (2.14)	−3.14 (2.14)	−1.60 (2.09)
**Region**							
rural (vs urban)		−5.45*** (0.95)	−5.06*** (0.95)	−6.33*** (0.89)	−4.62*** (0.99)	−5.76*** (0.97)	−5.36***(0.95)
**Income**							
lowest (vs. highest)			−5.01** (1.55)				−2.28 (1.50)
2nd (vs. highest)			−6.65*** (1.51)				−3.97**(1.46)
3rd (vs. highest)			−4.76** (1.47)				−3.09*(1.43)
4th (vs. highest)			−5.37*** (1.44)				−4.03**(1.40)
missing (vs. highest)			0.25 (1.86)				2.42-1.81
**Maternal PA**							
fairly (vs. very physically active)				−2.39*(1.17)			−1.99 (1.17)
not very (vs. very physically active)				−4.99***(1.40)			−4.28**(1.39)
not (vs. very physically active)				−3.09 (2.58)			−2.46 (2.57)
**Enrichment activities**				3.69***(0.56)			3.56***(0.56)
**Organised sport**				16.35***(0.90)			16.04***(0.90)
**Unstructured activities**				−0.02***(0.0)			−0.02***(0.00)
**School sporting facilities**							
excellent/good (vs. poor/fair)					−0.89 (0.89)		−0.96 (0.86)
**School commute**							
bus (vs. active commute)					−3.96** (1.32)		−3.92**(1.27)
car (vs. active commute)					−1.14 (1.14)		−1.77 (1.10)
**Perceiveddisorder**						−1.45* (0.65)	−0.81 (0.64)
**Perceivedsafety**						3.90*** (0.69)	2.74***(0.67)
**Constant**	111.28*** (1.81)	85.62*** (4.16)	87.43*** (4.18)	82.84*** (4.16)	86.74*** (4.24)	77.24*** (4.88)	78.42***(4.98)
**Observations**	8,962	8,962	8,962	8,962	8,962	8,962	8,962
**Number of id**	3,071	3,071	3,071	3,071	3,071	3,071	3,071
Controls	YES	YES	YES	YES	YES	YES	YES

Standard errors in parentheses.

***p < 0.001, **p < 0.01, *p < 0.05.

In Model 3, adolescents whose families belong to the highest income quintile were significantly more likely to be physically active than adolescents whose families belong to all other income quintiles, with one exception for males whose household belongs to the second highest (“4^th^”) income quintile. In Model 4, the family-level variables were associated with levels of PA in the expected direction. Adolescents whose mothers were “fairly” or “not very” or “not” active (compared to “very” active) were less physically active themselves. Participation in organised sport was significantly positively associated with levels of PA, and unstructured activities was significantly negatively associated with PA, for both males and females. Participation in enrichment activities had a significant positive effect on levels of PA for females and a non-significant negative effect on levels of PA for males.

In Model 5 the school-level mediating variables were related to PA in the expected direction, where adolescents with access to poor/fair sports facilities do less PA than those with access to good/excellent sports facilities, and active commuting (compared to travelling by bus or car) was positively associated with levels of PA. In model 6, living in a disorderly neighbourhood was significantly negatively associated with levels of PA for females. Unexpectedly, there was a small non-significant positive association between living in a disorderly neighbourhood and PA for males. Living in a safe neighbourhood was significantly positively associated with levels of PA for both males and females.

The final model is fully-adjusted for individual, material, family, school and neighbourhood factors. For males, having a mother who completed higher secondary (“leaving cert.”) compared to a tertiary education remains a significant negative predictor of PA at p < 0.05.

### 3.4 Decompositional analysis

Table 4 (below) provides the average difference (between ages 9, 13 and 17/18) in PA levels between young people whose mothers have lower secondary education (“junior cert.”) and young people whose mothers have tertiary education. This is referred to as the Maternal Education (ME) differential. Table 4 also provides the absolute reduction and the average proportionate reduction in PA with the addition of the each group of explanatory variables (income, family, school and neighbourhood), where model 2 (“controls”) provides the baseline ME differential. The models have been ranked by the level of change. The average proportionate change represents the contribution of each group of variables to explaining the social patterning of PA.

**Table 4:** Average proportionate change in the SEP differential in levels of PA with the addition of explanatory variables between age 9 and 17/18.

	Males			Females		
	**Average**	**Change**	**Change** **%**	**Average**	**Change**	**Change** **%**
Model 1: baseline	8.94	0.00	0.0%	13.98	0.00	0.0%
Model 5: school	7.72	0.10	1.2%	11.25	−0.01	−0.1%
Model 2: controls	7.82	0.00	0.0%	11.24	0.00	0.0%
Model 6: neighbourhood	7.58	−0.14	−1.5%	10.51	−0.73	−5.2%
Model 3: income	5.56	−2.16	−24.1%	9.19	−2.05	−14.7%
Model 4: family	3.18	−4.54	−50.8%	6.34	−4.91	−35.1%
Model 7: fully adjusted	2.19	−5.53	−61.8%	5.20	−6.05	−43.3%

The addition of household income to the model reduced the ME differential by 24.1% for males and 14.7% for females, making it the second most effective model at explaining the social patterning of PA.

The family model reduced the ME differential by 50.8% for males and 35.1% for females, making it the most effective model in explaining the social patterning of PA. In analysis (not shown) the unique contribution of each family-level variable was derived. Organised sport alone reduced the baseline ME differential by 43.6% for males and 25.3% for females, enrichment activities reduced the differential by 1.2% for males and 5.6% for females, unstructured activities reduced the differential by 18.6% for males and 14.8% for females and Maternal PA reduced the same differential by 2.8% for males and 2.5% for females. This analysis demonstrated that variables representing parenting practices (enrichment actives, organised sport and unstructured activities) were more effective in accounting for the gap in levels of PA between maternal education categories than the variables representing parents as role models (maternal PA).

The school model did not contribute to the explanation of ME differentials in PA, indeed for males the ME differential increased in model 5 by 1.2%. There was a modest reduction for females of 0.1%. This may be due to the reverse social patterning of the school commute variable, where families with low levels of maternal education were more likely to have an active commute (as outlined in the previous section). Adjusting for neighbourhood-level characteristics had a small impact on ME differential, reducing it by 1.5% for males and 5.2% for females.

The fully adjusted model, including individual, family, school and neighbourhood characteristics reduced the ME differential by 61.8% for males and 43.3% for females (average of three timepoints). Our models were better able to account for the socially patterning of PA for males compared to females. Figs. 3 and 4 (see graphs below) visually displays the reduction of the ME differential with the addition of explanatory variables at age 9, 13 and 17/18 separately.

**Graph 2:**
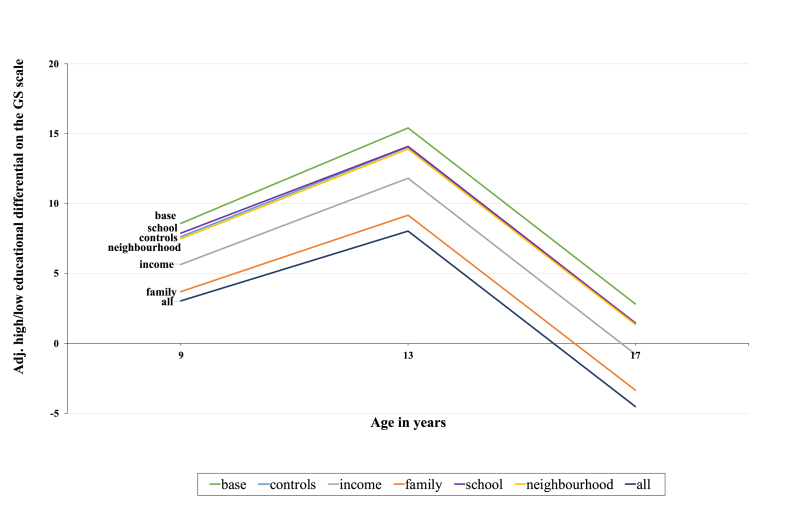
Estimated high/low educational differentials in levels of PA on the GS scale (males).

**Graph 3:**
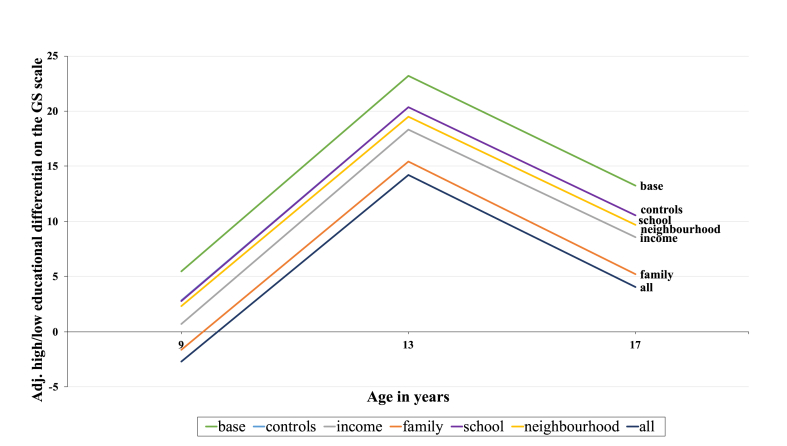
Estimated high/low educational differentials in levels of PA on the GS scale (females).

## 4 Conclusion

Physical activity is one of the common modifiable risk factors for the emergence of NCDs amoung low SEP groups. This paper aimed to understand the role of family, school and neighbourhood-level processes in accounting for the maternal education gradient in PA among adolescents between ages 9 and 18 using a large, nationally representative sample from the Republic of Ireland. Using a decompositional approach, we quantified the contribution of family, school and neighbourhood-level factors adjusting for individual-level personality, BMI and health-status and household-level income.

Our data are from Ireland, but our descriptive findings support evidence from other national contexts that show a strong association between social and economic position and PA (Inchley et al., 2016, 2020) and a sharp decline in PA during adolescence (Dumith et al., 2011). Internationally there is variation in the age at which the decline in PA occurs during adolescence, but the literature suggests somewhere between 11 and 15yrs. Given the data points available to us, it was only possible to be certain that PA peaked before the age of 13 but may have been decreasing since age 9. Young people with lower educated mothers experienced steeper declines in PA than their more advantaged peers over the ten-year period of observation.

We looked at the potential role of equivalised family income in mediating the relationship between maternal education and adolescent PA. Adjusting for income, our decompositional analysis accounted for 24.1% of the ME differential in levels of PA for males, and 14.7% for females. The ME differential was further reduced by the addition of family, school and neighbourhood level factors, suggesting that there were also cultural and social mechanisms through which maternal education influences young people's PA.

### 4.1 Family-level mechanisms

Family-level mechanisms accounted for the biggest reduction in the ME differential in PA for both males and females, they included: enrichment activities (youth club, dance, drama, music, organised sport etc.), unstructured activities (playing video games, time on the computer a, watching TV) and maternal levels of PA. Considered separately, parents enrolling their children in organised sport was the most effective family-level mechanism for explaining the social patterning of PA. The proportionate reduction was larger for males compared to females, which is in-line with Lareau's (2011) observation that her male participants were more likely to opt for organised sport (e.g. basketball, baseball, and soccer) as their primary enrichment activity, while female enrichment activities were more varied. Participation in organised sport has already been shown to account for both the age decline (Lunn et al., 2013; Lunn & Layte, 2008; Zick et al., 2007) and gender differences (Lunn et al., 2013; Vilhjalmsson & Kristjansdottir, 2003) in levels of PA. Our findings indicate that participation in organised sport also contributes to the social patterning in levels of PA. Recent Irish qualitative research identified barriers to organised sport specific to low education families, including: not feeling welcome, expense and transportation (Tandon et al., 2021).

As shown elsewhere (McCoy et al., 2012), and in line with Lareau's theory of “natural growth” (2011), we found that adolescents of lower educated mothers were more likely to partake in unstructured activities, and this contributed significantly to the social patterning of PA. This would support a “displacement hypothesis” (Pearson et al., 2014) where engagement in sedentary activities displaces time that would have otherwise been spent being physically active.

This paper also investigated whether parents engaged in sport and PA acted as role-models for their children. We found that adjusting for maternal PA levels reduced the ME differential for both males and females, making it an important part of the intergenerational transmission of inequalities in levels of PA in the home. However, our decompositional analysis revealed that controlling for parenting practices, compared to parental role-modelling, was more effective in accounting for the social patterning of PA.

### 4.2 School-level mechanisms

We did not find significant differences in the availability of school sporting facilities to young people in the different maternal education categories, which was unexpected given the qualitative reporting in the Irish context (Woods et al., 2010, 2019). Additionally, our analysis showed that active commute to school was actually more common among adolescents whose mothers had lower levels of education when compared to their more advantaged peers. This reverse social patterning has previously been documented in adolescents in Spain, Canada and Australia (Carlin et al., 1997; Chillón et al., 2010; Robertson-Wilson et al., 2008). Previous research, in the Irish context, reported that active commuting is more likely in urban settings (Murtagh et al., 2016). This suggests that our results may be influenced by the high concentration of lower SEP families in urban areas in Ireland.

The school environment did not contribute to the reduction in ME differential in levels of PA but it is possible that other, unmeasured, school-level processes may have an influence on levels of PA that were not captured in this study. Moreover, our dependent variable (the GS scale) is the parent's estimate of their child's PA. Given this, it is perhaps not surprising that variation in the sports which parents organise is significant in our models, whilst sport played in school, which parents were probably less likely to see, is not significant.

### 4.3 Neighbourhood-level mechanisms

Drawing on the socio-ecological models of health behaviours (Sallis et al., 2015) and answering the call for a focus on sub-groups within this area of research (Biddle & Mutrie, 2007), we argued that the association of higher education with neighbourhoods with safe spaces to exercise and low-levels of crime, vandalism, litter etc. may account for some of the social patterning in PA. Adjusting for perceived level of neighbourhood safety and neighbourhood disorder produced a small reduction in the ME differential in levels of PA for males and females. The proportionate reduction was larger for females, compared to males, which suggests that neighbourhood safety and disorder is a bigger barrier to PA for lower SEP females. Our evaluation of the young person's physical environment was limited by the measures available in our chosen dataset. Adjusting for other features of neighbourhood environment could elevate the role of neighbourhood in explaining the social patterning of PA.

### 4.4 Strengths and limitations

Research on the social distribution of PA is often based on small, non-representative samples, partly because reliance on data from accelerometers, which is expensive and time consuming to collect. Although our dependent variable was based on parental report, the study provides information on a nationally representative sample of young people covering an observation period from middle childhood to early adulthood. Combined with the wide variety of dimensions available, this enabled us to carry out a multi-dimensional longitudinal study. However, our use of a parent-reported PA is a clear limitation. Accelerometer data are not subject to respondent bias or lapses in memory, or other limitations associated with self-report measures. However, the Godin-Shepherd is an internationally validated instrument (Godin et al., 1986; Godin & Shephard, 1985; Zelener & Schneider, 2016). Although the absolute level of PA reported may not be as accurate as that derived from accelerometer data, it seems reasonable to assume that any biases involved are equal across groups defined by maternal level of education. The focus of this research was the relative differences between ME groups rather than the absolute levels of PA in each group.

#### Research Ethics

The study received ethical approval from the Research Ethics Committee of the Office for the Minister for Children and Youth Affairs in Ireland. This study reports results from analysis of deidentified publicly available survey data and is exempt from institutional review board review as per section 46.10(b) of National Institutes of Health document CFR 46.

## Financial disclosure

This research is funded by the Department of Sociology, Trinity College Dublin (TCD).

The Role of Family, School and Neighbourhood Processes in Explaining Inequalities in Physical Activity Trajectories Between age 9 and 18.

## CRediT authorship contribution statement

**Olivia McEvoy:** Conceptualization, Data curation, Methodology, and analysis, Writing – original draft. **Frances Cronin:** Methodology, and analysis. **Ross Brannigan:** Methodology, and analysis. **Debbi Stanistreet:** Writing – review & editing. **Richard Layte:** Supervision.

## Declaration of competing interest

Authors have no conflicts of Interest to declare.

## Data Availability

Data will be made available on request.
